# Draft Genome Sequence of Rhodotorula mucilaginosa from an Adult Patient in Qatar

**DOI:** 10.1128/MRA.00725-21

**Published:** 2021-10-21

**Authors:** Sathyavathi Sundararaju, Husam Salah, Emad B. Ibrahim, Andres Perez-Lopez, Fatma Ben Abid, Clement K. M. Tsui

**Affiliations:** a Department of Pathology, Sidra Medicine, Doha, Qatar; b Division of Microbiology, Department of Laboratory Medicine and Pathology, Hamad Medical Corporation, Doha, Qatar; c Weill Cornell Medicine-Qatar, Doha, Qatar; d Department of Medicine, Division of Infectious Diseases, Hamad Medical Corporation, Doha, Qatar; e Division of Infectious Diseases, Faculty of Medicine, University of British Columbia, Vancouver, Canada; University of California, Riverside

## Abstract

Rhodotorula mucilaginosa is an opportunistic fungal pathogen of public health importance. We present the draft genome sequence of an isolate (Rhodo3571) cultured from an immunocompetent patient. The isolate is similar to other *R. mucilaginosa* genomes in the NCBI database. Presented here are the genome assembly and its comparison to other reference genomes.

## ANNOUNCEMENT

*Rhodotorula* is a pigmented yeast, which is a normal environmental/commensal microorganism but it can cause opportunistic infections, such as those of the bloodstream, meningitis, and peritonitis (1, 2). Rhodotorula mucilaginosa has emerged as an opportunistic etiologic agent, particularly in immunocompromised patients, and infections have been reported in different parts of the world (3–6), including a recent *Rhodotorula* fungemia reported in Qatar (7). This article does not contain any studies with human participants or animals performed by any of the authors.

Here, we present a draft genome assembly of *R. mucilaginosa* (Rhodo3571) from an immunocompetent host, possibly associated with central venous catheter (CVC) infection at Hamad Medical Corporation (Doha, Qatar) (7). The yeast was isolated from blood; the blood culture aerobic vial that was flagged positive was subcultured on Sabouraud’s dextrose agar (SDA; Difco, USA) and incubated under aerobic conditions at 35 ± 2°C for 18 to 24 h, minimizing exposure to light. After incubation, pink colonies were isolated on SDA, and the isolate was identified by matrix-assisted laser desorption ionization–time of flight mass spectrometry (MALDI-TOF MS) using the Bruker microflex system version 4 and the MALDI Biotyper (MBT) BDAL version 9.0 library (Bruker Daltonics, Germany). The identification was performed using the extended direct transfer method recommended by Bruker for identification of yeasts. In brief, a uniform thin layer of a yeast colony was smeared onto the MALDI target plate and overlaid with 1 μl 70% formic acid. It was allowed to dry at room temperature; then, 1 μl of HCCA matrix (Bruker Daltonics) was added to it. It was allowed to dry at room temperature again and then measured using the Bruker microflex system. The isolate was identified as *R. mucilaginosa* with a score of 2.08, which is considered reliable genus and species identification according to Bruker’s cutoff score of ≥2.00. Antifungal susceptibility patterns were also characterized (7).

After growing the culture on SDA for 2 days, genomic DNA was extracted using the MasterPure yeast DNA purification kit (Lucigen Corporation, WI, USA). The DNA concentration was measured using the Qubit 2 fluorometer (Thermo Fisher), and DNA libraries were constructed using the Nextera XT DNA library preparation method (Illumina, Inc., CA, USA) and sequenced on the Illumina NextSeq 550 platform with 300 cycles (150-bp paired-end format) at Sidra Medicine. The adapter sequences were removed, and low-quality bases were trimmed from the raw reads using Trim Galore v.0.6.5 (http://www.bioinformatics.babraham.ac.uk/projects/trim_galore/). The read quality was confirmed using FastQC v.0.11.9 (https://github.com/s-andrews/FastQC). The reads were assembled using SPAdes v.3.12.0 (8). Small contigs (<1,000 bp) were discarded, and the assembly statistics were estimated using QUAST v.5.0.2 (9). Parsnp v.1 (10) was used to infer the genetic relationships among other *R. mucilaginosa* genome assemblies in GenBank; single-nucleotide polymorphisms (SNPs)/variants detected from the core genome alignment were used to infer the phylogenetic relationship using RAxML, implemented in Parsnp. Default parameters were used for all software, unless otherwise specified.

**FIG 1:**
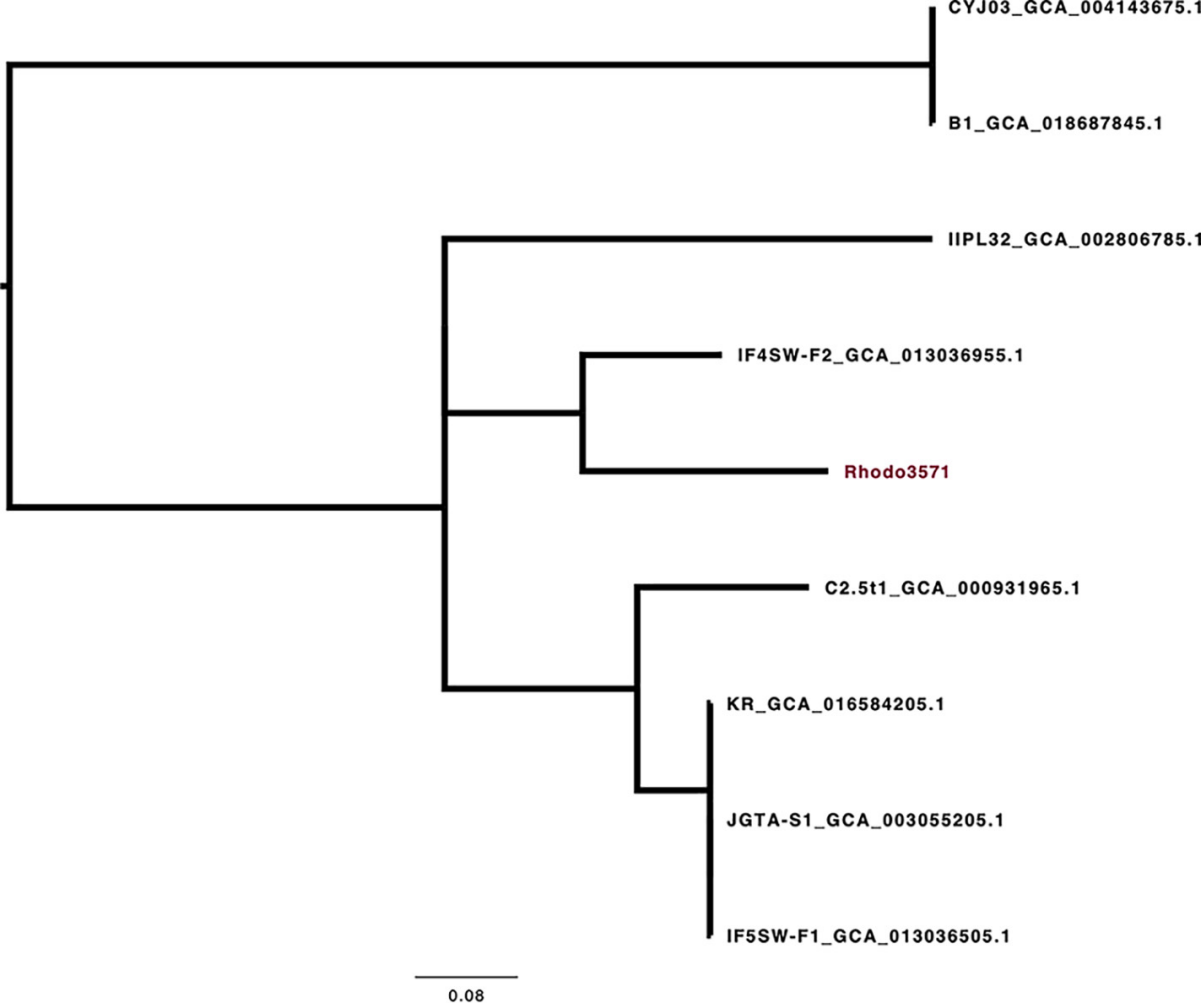
Genetic relationship among *R. mucilaginosa* available genomes based on core genome SNPs (rooted at the midpoint).

The genome statistics and information are summarized in Table 1. The genome sequence based on *k*-mer 55 contained 789 contigs, with an *N*_50_ value of 49,539 bp. Rhodo3571 is an important clinical sample in Qatar; phylogenetic analysis of selected environmental isolates showed that Rhodo3571 was most related to strain IF1SW-B1 (GenBank accession number GCA_013036955.1), a strain recovered from the indoor biome in the International Space Station (Fig. 1). Since *R. mucilaginosa* is a rare and difficult-to-treat pathogen, the draft genome sequence will contribute to further investigation into the molecular mechanisms that lead to pathogenesis and antifungal drug resistance.

**TABLE 1:** Rhodotorula mucilaginosa genome statistics and information

Characteristic	Data
Organism	Rhodotorula mucilaginosa Rhodo3571
BioProject accession no.	PRJNA743597
BioSample accession no.	SAMN20056676
Total no. of reads	4,659,413
Coverage (×)	70
Genome length (bp)	19,947,800
*N*_50_ (bp)	49,539
No. of contigs	789
GC%	60.55

### Data availability.

The whole-genome shotgun data from this study have been deposited in the DDBJ/ENA/GenBank repositories under accession numbers PRJNA743597 and JAHUZI000000000. The version described in this paper is version JAHUZI010000000.
